# Accuracy of patient-specific CT organ doses from Monte Carlo simulations: influence of CT-based voxel models

**DOI:** 10.1007/s13246-024-01422-z

**Published:** 2024-04-18

**Authors:** Gwenny Verfaillie, Jeff Rutten, Yves D’Asseler, Klaus Bacher

**Affiliations:** 1 Department of Human Structure and Repair, Ghent University, Ghent, Belgium; 2 Department of Nuclear Medicine, Ghent University Hospital, Ghent, Belgium; 3 Department of Diagnostic Sciences, Ghent University, Ghent, Belgium

**Keywords:** Computed Tomography (CT), Monte Carlo, Patient-specific dosimetry, Organ dose

## Abstract

Monte Carlo simulations using patient CT images as input are the gold standard to perform patient-specific dosimetry. However, in standard clinical practice patient’s CT images are limited to the reconstructed CT scan range. In this study, organ dose calculations were performed with ImpactMC for chest and cardiac CT using whole-body and anatomy-specific voxel models to estimate the accuracy of CT organ doses based on the latter model. When the 3D patient model is limited to the CT scan range, CT organ doses from Monte Carlo simulations are the most accurate for organs entirely in the field of view. For these organs only the radiation dose related to scatter from the rest of the body is not incorporated. For organs lying partially outside the field of view organ doses are overestimated by not accounting for the non-irradiated tissue mass. This overestimation depends strongly on the amount of the organ volume located outside the field of view. To get a more accurate estimation of the radiation dose to these organs, the ICRP reference organ masses and densities could form a solution. Except for the breast, good agreement in dose was found for most organs. Voxel models generated from clinical CT examinations do not include the overscan in the z-direction. The availability of whole-body voxel models allowed to study this influence as well. As expected, overscan induces slightly higher organ doses.

## Introduction

Computed tomography (CT) plays an important role in medical imaging. New techniques, protocols and technologies make it not only suitable for diagnostic imaging but also for screening of lung and colon cancer, and to guide minimally invasive interventional procedures. In addition, its use in hybrid nuclear medicine imaging (PET/CT and SPECT/CT) is growing. Therefore, the number of CT examinations has increased over the past decades. This upward trend is not confined to the European countries. The NCRP reported an increase by 20% in the number of CT scans performed in the United States over the decade 2006–2016 [1, 2]. However, the annual average effective dose per inhabitant from CT remained stable [1, 3]. Although the frequency of performed CT examinations is small compared to other modalities, up to 64% of the radiation dose in medical imaging is still delivered by CT [1, 3–7]. The growing concern about the long-term effects of radiation exposure, especially the risk of cancer, increased the need to have accurate patient dose estimates [7, 8].

The frequently used dose indicators are volume CT dose index (CTDI_vol_) and dose-length product (DLP), which are determined as standard to a 16 or 32 cm diameter IEC CT dosimetry phantom [9]. To incorporate the patient’s size, the AAPM Task Groups 204 and 220 [10, 11] introduced the effective and water equivalent diameter metric, respectively. Scaling CTDI_vol_ according to this methodology results in a size-specific dose estimate (SSDE). Nevertheless, to assess potential radiation risks associated with CT exposure, accurate individual organ dose estimations are needed. For this purpose, easy-to-use dose calculation tools such as CT-Expo [12] and NCICT [13] were developed. However, these tools may have some limitations. For instance, the number of available phantoms can be limited or automatic tube current modulation cannot accurately be applied. Therefore, dedicated Monte Carlo (MC) dose simulations using patient-specific voxel geometries, which give a realistic representation of the patient’s body, are needed [14]. These individualised 3D voxel models can be created based on clinically available CT data of the patient.

In conventional CT and hybrid nuclear medicine, the available clinical CT data is limited to the scan range that most of the times does not cover the patient’s whole body. This influences the accurate incorporation of scatter from the rest of the body and the dose estimation of organs lying outside or partially in the imaged volume [2, 8]. Secondly, voxel models generated from clinical CT examinations do not include the overscan in the superior-inferior direction, the z-direction, which may result in a slight underestimation of calculated organ doses [15]. Organ doses obtained through Monte Carlo simulations based on this limited data must thus be taken with caution. Although some studies acknowledge the limitations of a voxel model limited to the clinical CT scan range, almost none of them studied the accuracy of organ doses obtained with it.

The purpose of this study was to estimate the accuracy of patient-specific organ doses of chest and cardiac CT scans through Monte Carlo simulations based on voxel models limited to the specific CT scan range and whole-body voxel models. In addition, the influence of overscan was investigated.

## Materials and methods

### Patients and voxel models

Whole-body CT images of fifty adult patients, acquired during a whole-body PET/CT examination on a 40-slice Siemens Biograph mCT Flow (Siemens Healthineers, Germany), were collected retrospectively. They were chosen in such a way to assure a wide variety in Body Mass Index (BMI) (Table 1). An equal number of male and female patients was selected. To be suitable for accurate dose estimations, the reconstructed Field of View (FOV) of the CT scans included the entire cross-section of the patient. The retrospective use of the CT images was approved by the institutional ethical committee. All images were selected and extracted from the institutional Picture Archiving and Communication System (PACS). To comply with the current General Data Protection Regulation (GDPR) rules, all CT data was anonymised according to the hospital’s anonymisation policy before extraction from the PACS. Only data concerning patient sex, age, length and weight was kept.

**Table 1:** Summary of mean (minimum–maximum) age, length, weight and BMI of the study population

Study population	Age (years)	Length (m)	Weight (kg)	BMI (kg/m^2^)
25 females	57 (24–86)	1.62 (1.50–1.74)	65 (39–94)	25 (15–36)
25 males	64 (33–84)	1.76 (1.60–1.95)	79 (51–126)	26 (16–35)

Based on the data of the 512 × 512 DICOM images, a patient-specific 3D whole-body voxel model was created for each patient with 0.9727 × 0.9727 × 3 mm^3^ voxel size.

**Fig. 1:**
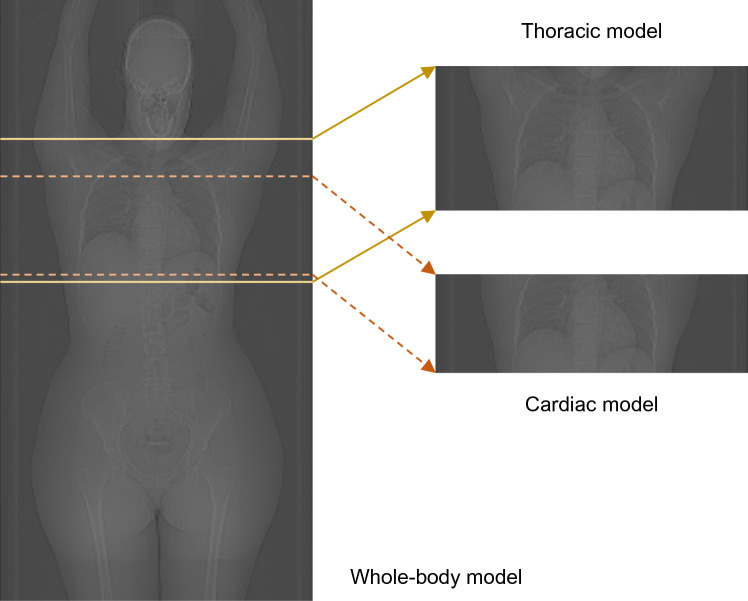
Clinical whole-body CT images were used to create patient-specific 3D voxel models: a whole-body, a thoracic and a cardiac model

In standard clinical practice, the available image data are limited to the patient’s CT scan range, and no information exists about the rest of the body. Therefore, two additional voxel models were created for each patient. One using only the thoracic region of the original whole-body voxel model and a second using only the images of the cardiac region (Fig. 1).

### Monte Carlo dose simulations

To estimate patient-specific CT organ doses, Monte Carlo (MC) simulations were performed with ImpactMC 1.6 (CT Imaging GmbH, Erlangen Germany), a validated patient-specific dose calculation tool [16–19]. It combines Monte Carlo algorithms with scanner specific parameters such as geometric, spectral and shaped filter characteristics, and patient-specific voxel models based on patient CT images. In this way, the software calculates individualised 3D dose distributions, considering all relevant photon interaction processes [16, 17]. Delineation of the organs of interest makes it then possible to estimate patient-specific organ and tissue doses.

In this study, the CT part of a Siemens Biograph mCT Flow PET/CT (Siemens Healthineers, Germany) was modelled. Geometrical specifications, such as the focus to isocenter distance (595 mm) and fan angle (0.7955), were derived from specific data elements, DICOM tags, in the DICOM header of the CT images. However, they could also be extracted from the technical reference manual of the system. To specify the X-ray spectrum, the methodology described by Turner et al*.* [20] for equivalent energy spectra in CT was used. Based on experimental derivation of the first half-value layer, an equivalent spectrum was generated with a MATLAB code (Mathworks, USA) with added SPEKTR tool [21, 22]. The bowtie filter profile was characterised based on dose measurements. For this, a calibrated pencil beam ionisation chamber (Model 10X6-3CT, Radcal Corporation, USA) was moved in 1 cm intervals from the isocenter while keeping the X-ray tube stationary [20]. In addition, the air kerma free-in-air in the isocenter of the CT was measured since the calibration of the simulation software is based on it.

Chest and cardiac CT examinations were simulated using both the adjusted voxel model, limited to the corresponding anatomical scan range, and the whole-body voxel model. All simulations were performed using the scan parameters of a diagnostic whole-body CT embedded in the DICOM header of the original whole-body voxel models. Helical scans were simulated at 120 kV with a rotation time of 0.5 s, a beam collimation of 19.2 mm and a pitch of 0.7 (Table 2). Tube current modulation (TCM) is available on most CT scanners and is employed for most clinical protocols. The TCM system available on the simulated CT scanner is CARE Dose4D. Therefore, the tube current value in the DICOM header of each reconstructed slice is the average of the applied angularly and longitudinally modulated values [23–28]. To integrate TCM in the Monte Carlo simulation software ImpactMC, these average tube current values were extracted from the DICOM header of each reconstructed image using an in-house developed Fiji/ImageJ macro. For the chest CT scan, the simulation started and ended at the lung apex and base, respectively. For the cardiac CT scan, the scan range was defined from the aortic arch to the heart apex. Finally, 3D dose distributions were obtained by simulating the interactions and dose depositions of a large number of photons. To ensure the speed and accuracy of the Monte Carlo simulation, the number of interacting photons was chosen to be 10^10^ for all simulations.

**Table 2:** Summary of exposure parameters for chest and cardiac CT examinations

Parameter	Chest	Cardiac
Tube voltage (kV)	120	120
Tube current (mA)	ATCM*	ATCM*
Rotation time (s)	0.5	0.5
Pitch	0.7	0.7
Beam collimation (mm)	19.2	19.2
Scan FOV (mm)	500	500
Scan start	lung apex	aortic arch
Scan end	lung base	heart apex

*Automatic Tube Current Modulation

In addition, the effect of overscan was investigated. Using the whole-body voxel model, Monte Carlo dose simulations of a chest and cardiac CT scan were performed considering overscan as an extension of the original chest or cardiac scan range. In this study, we assumed an overscan of 12 mm in both the superior and inferior direction of the original scan range. All other simulation parameters were kept the same.

### Organ dose calculation

#### Delineation of organs

The radiosensitive organs and tissues of interest were delineated on the original whole-body CT images by a medical physicist. These regions of interest (ROIs) were obtained semi-automatically for the lungs, bones (ribs/spine) and liver while for the breast (female patients), heart, kidneys, thyroid and oesophagus manual delineation was performed. For this, the open source software tools Fiji/ImageJ [29, 30] and 3D Slicer [31] were used.

#### Patient-specific organ doses

A Monte Carlo dose calculation with ImpactMC results in a 3D dose distribution based on the physical properties (i.e., attenuation, composition and size) of the input patient CT scan. Overlaying the contours of each organ on the corresponding slices of the dose distribution results in an estimation of patient-specific organ doses *D*_*T*_ which were determined as follows:

$${D}_{T}=\sum_{i=1}^{N}\left({f}_{i,T}\cdot {M}_{i,T}\right) \,with \,{f}_{i,T}=\frac{{A}_{i,T}}{{\sum }_{i=1}^{N}{A}_{i,T}}$$

where *M*_*i,T*_ is the mean dose within the contour at slice *i* of organ *T*, *N* the total number of slices that contain contours of organ *T* and *f*_*i,T*_ the fractional area of each organ contour (with *A*_*i,T*_ the area within the contour at slice *i* of organ *T*).

To enable unsupervised organ dose calculation, an algorithm was implemented in Fiji/ImageJ. Estimations of organ doses normalised to the mean tube current–time product (mAs) were determined for chest and cardiac CT examinations simulated using both the anatomy-specific and whole-body voxel models. Because the total organ volume of all organs of interest is known from the whole-body segmentations, the organ doses resulting from the simulations with the adjusted voxel models were recalculated. In this way, the influence of missing volumetric information of organs that are partially out of the field of view could be studied. Finally, normalised organ doses were calculated for the Monte Carlo simulations of chest and cardiac CT scans that included the principle of overscan.

#### Reference organ volumes

In standard clinical practice, most CT scans do not contain whole-body information. To get a more accurate estimation of the dose of organs lying partially in the field of view reference organ volumes could be used instead. These were calculated according to the ICRP 89 reference organ masses and ICRP 110/145 reference organ densities for the reference adult male and female (Table 3) [32–34]. With these reference volumes, the organ doses *D*_*T*_ obtained from simulations using the anatomy-specific voxel model were recalculated as follows:

$${D}_{T, ref volume}={D}_{T}\cdot \frac{{V}_{T}}{{V}_{T, ref}}$$

where *V*_*T*_ en *V*_*T, ref*_ are the organ volume present in the voxel model and reference organ volume, respectively.

**Table 3:** ICRP reference organ masses (ICRP 89 [32]) and densities (ICRP 110 [33] and ICRP 145 [34]) for the reference adult male (1.76 m; 73 kg) and female (1.63 m; 60 kg)

	Reference adult male			Reference adult female		
Organ	Mass (kg)	Density (kg/m^3^)	Volume (m^3^)	Mass (kg)	Density (kg/m^3^)	Volume (m^3^)
Breast	0.025	1020	2.45 × 10^–5^	0.500	1020	4.90 × 10^–4^
Liver	1.800	1060	1.70 × 10^–3^	1.400	1060	1.32 × 10^–3^
Lungs^a^	1.200	415	2.89 × 10^–3^	0.950	413	2.30 × 10^–3^
Kidneys^b^	0.217	1050	2.07 × 10^–4^	0.193	1050	1.83 × 10^–4^
Oesophagus	0.040	1037	3.86 × 10^–5^	0.035	1036	3.38 × 10^–5^
Ribs^c^	0.735	1350	5.44 × 10^–4^	0.437	1350	3.24 × 10^–4^
Thyroid	0.020	1051	1.90 × 10^–5^	0.017	1051	1.62 × 10^–5^
Spine^c^	1.995	1350	1.48 × 10^–3^	1.591	1350	1.18 × 10^–3^

^a^Including pulmonary and bronchial blood

^b^Only cortex

^c^Fraction of the total skeletal mass (male: 10.5 kg; female: 7.8 kg) (various bones, including bone marrow): ribs (male: 7%; female: 5.6%), spine (male: 19%; female: 20.4%)

### Comparison of organ dose estimations

When using anatomy-specific voxel models, information related to helical overscan and the rest of the body is missing. The availability of whole-body voxel models offers the opportunity to study the influence of particular modelling deficiencies. For both the thoracic and cardiac CT scan, organ dose estimations obtained through Monte Carlo simulations performed using the whole-body voxel model and including overscan represent the best obtainable estimate of truly received organ doses to the patient. Comparing these organ doses with those acquired from simulations applying the anatomy-specific voxel models, yields information about missing organ volumes as well as scatter from the rest of the body and overscanning (Comparison C4). To get an idea of the effect of overscan, the organ doses estimated from simulations using the whole-body voxel model with and without the inclusion of overscan were compared with each other (Comparison C3). The influence of missing organ volume was determined by comparing the original organ doses from the simulations using the anatomy-specific voxel models and those recalculated considering the entire organ volume (Comparison C1). These recalculated doses were also compared to the truly received organ doses, yielding information about both scatter from the rest of the body and overscan (Comparison C2). For each comparison the mean percentage difference in organ dose and its standard deviation was calculated. Organ dose differences were also determined between the truly received organ doses and the doses estimated applying the anatomy-specific voxel model but recalculated with the reference organ volumes (Comparison C5).

In addition, the correlations between the contribution of scatter from the rest of the body and the organ volumes with patient characteristics such as BMI, weight and water equivalent diameter were investigated through regression analysis. The coefficient of determination, R^2^, was used as a measure to assess the strength of the correlation.

## Results

**Fig. 2:**
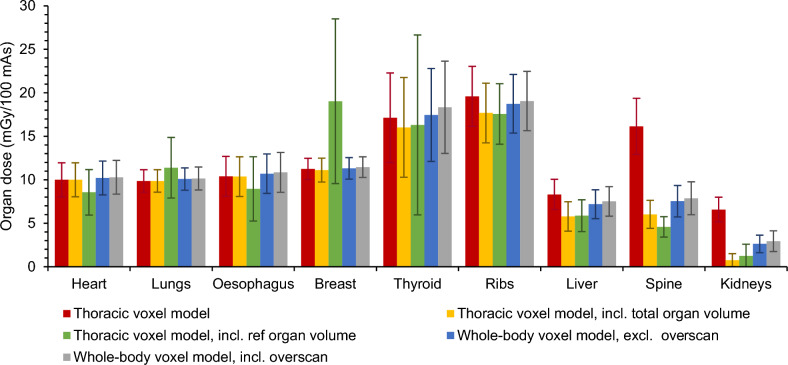
Monte Carlo simulated mean organ doses of a chest CT scan at 120 kV with tube current modulation on a Siemens Biograph mCT Flow PET/CT using the thoracic voxel model, without and with taking into account the total or reference organ volume of partially irradiated organs, and the whole-body voxel model without and with overscan

**Fig. 3:**
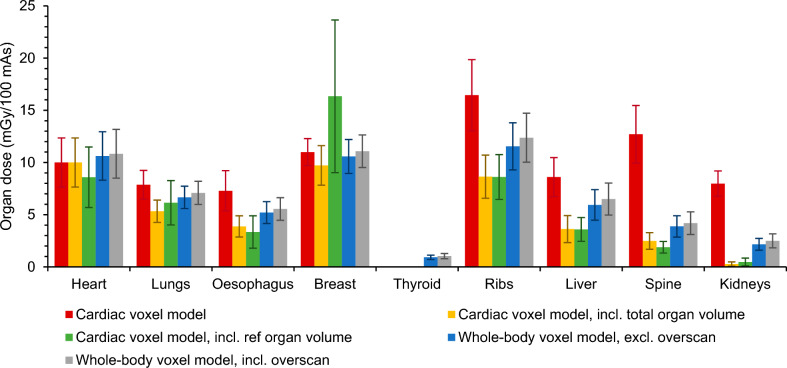
Monte Carlo simulated mean organ doses of a cardiac CT scan at 120 kV with tube current modulation on a Siemens Biograph mCT Flow PET/CT using the cardiac voxel model, without and with taking into account the total or reference organ volume of partially irradiated organs, and the whole-body voxel model without and with overscan

Organ dose calculations were performed for the heart, lungs, oesophagus, breast, thyroid, ribs, liver, spine and kidneys which are all located completely or partially in the thoracic region. For all patients, estimated normalised mean organ doses and their standard deviations are displayed in Fig. 2 and Fig. 3 for, respectively, chest and cardiac CT scans simulated using both the thoracic or cardiac and whole-body voxel models.

When using the anatomy-specific voxel model, considering the entire organ volume leads to a reduction in dose estimates for all organs lying partially outside the field of view. For a chest CT, the largest dose decreases are observed for the kidneys (−89%) and the spine (−63%) while the dose for the liver lies only 30% lower (Fig. 2). Smaller differences are seen for the ribs (−10%), thyroid (−6%), breast (−1%) and oesophagus (−0.3%) which lie almost completely in the field of view. No dose differences are observed for the heart and lungs because the CT scan range covers them entirely. Similar results are found for a cardiac CT where dose decreases around 97%, 80%, 58%, 47%, 47%, 32% and 12% are found for the kidneys, spine, liver, ribs, oesophagus, lungs and breast, respectively (Fig. 3). No conclusion could be drawn for the thyroid because it is not in the scan area. For most organs, little to no correlation (0 ≤ R^2^ ≤ 0.3) is found between the real organ volume and patient characteristics like BMI, weight and water equivalent diameter while for the breast a weak to moderate correlation (0.35 ≤ R^2^ ≤ 0.55) is seen.

Next, scatter from the rest of the body can be incorporated as well when using whole-body voxel models. Compared to the previous situation in which only the entire organ volume is taken into account, considering scatter from the rest of the body leads to a dose increase for all organs. In chest CT, these increases are rather small (≤ 4%) for organs located (almost) completely in the field of view, such as the heart, lungs, oesophagus and breast. For the ribs and thyroid, this increase is around 6% to 9% while the largest dose increases are observed for the spine, liver and kidneys. Similar results are found in cardiac CT. There, organ dose increases of around 6% to 9% are observed for the heart and breast, respectively. Organs located more outside the field of view, like the lungs, ribs, oesophagus, spine and liver, show a dose increase ranging from 25% over 34% to 64% while an even higher increase is seen for the kidneys. As can be seen in Fig. 3, organ doses for the thyroid can now be calculated as well. However, it is important to notice that only scatter contributes to the thyroid dose. Through regression analysis, little to no correlation (0 ≤ R^2^ ≤ 0.3) was found between the contribution of scatter and BMI or water-equivalent diameter.

### Influence of the CT-based voxel model

Tables 4 and 5 present the percentage differences in mean organ doses between Monte Carlo dose calculations performed with the anatomy-specific and whole-body voxel models for chest and cardiac CT, respectively. When the adjusted voxel model is used, radiation doses of organs that are partially outside the field of view are overestimated when the entire organ volume is not considered (Tables 4 and 5–C1). For organs of which a larger percentage of the volume is situated outside the primary exposed volume, the overestimation is the largest. Compared to organ doses estimated using the whole-body voxel model and incorporating overscan, organ doses obtained using the adjusted voxel model but recalculated for the entire organ volume underestimate the organ doses received by the patient because now only scatter from the rest of the body and overscan is ignored (Tables 4 and 5–C2). Overall, if patient data is limited to the CT scan range, organ doses of organs that are (almost) completely in the field of view are underestimated while for all other organs, the organ dose is overestimated (Tables 4 and 5–C4). In chest CT, breast, heart, lung, thyroid and oesophagus dose are underestimated by around 1.8% to 7% (Table 4–C4). The rib, liver, spine and kidney doses on the other hand are overestimated by around 3%, 12%, 108% and 149%, respectively. To get an idea on how these under- and overestimations of organ doses are reflected in absolute dose values, the mean tube current–time product of the chest CT scans in this study of 141 mAs, which corresponds well with typical values found in literature, was applied [35, 36]. Organ doses of 13.9 ± 1.8 mGy, 11.7 ± 2.5 mGy and 9.3 ± 2.0 mGy are found for the lungs, liver and kidneys, respectively, when using the thoracic voxel model. When the whole-body voxel model is used instead, dose values of 14.2 ± 1.8 mGy, 10.1 ± 2.3 mGy and 3.7 ± 1.4 mGy are found for, respectively, the lungs, liver and kidneys. In case of a cardiac CT, the heart dose is underestimated by around 8% while for all other organs, except the thyroid and breast, the organ dose is overestimated. For the thyroid, the dose cannot be estimated when it is not imaged in the voxel model. Because only scatter contributes to the thyroid dose, an underestimation of 100% is found.

**Table 4:** Percentage difference in mean organ dose and standard deviation for a chest CT scan at 120 kV with tube current modulation of: C1–A chest CT using the thoracic voxel model compared to a chest CT using the same model but taking into account the entire organ volume, C2–A chest CT using the thoracic voxel model but taking into account the entire organ volume compared to a chest CT with overscan using the whole-body voxel model, C3–A chest CT with overscan compared to a chest CT without overscan using the whole-body voxel model, C4–A chest CT using the thoracic voxel model compared to a chest CT with overscan using the whole-body voxel model

	Organ dose difference (%)			
Organ	C1	C2	C3	C4
Heart	0.0 ± 0.0	−2.9 ± 1.9	0.8 ± 1.2	−2.9 ± 1.9
Lungs	0.0 ± 0.0	−2.8 ± 0.9	0.6 ± 0.9	−2.8 ± 0.9
Oesophagus	0.3 ± 1.0	−4.7 ± 2.1	1.4 ± 1.3	−4.4 ± 1.7
Breast	1.7 ± 8.7	−3.1 ± 5.5	1.4 ± 2.8	−1.8 ± 2.5
Thyroid	12.1 ± 29.9	−14.1 ± 12.2	5.8 ± 5.6	−6.9 ± 4.6
Ribs	11.4 ± 6.7	−7.5 ± 2.6	1.7 ± 1.6	2.9 ± 3.9
Liver	51.7 ± 43.3	−23.9 ± 9.3	4.9 ± 5.9	11.9 ± 14.0
Spine	174.7 ± 36.2	−24.0 ± 2.9	4.6 ± 2.0	108.2 ± 22.7
Kidneys	3929.2 ± 7025.4	−78.9 ± 14.3	12.4 ± 13.7	148.7 ± 86.8

**Table 5:** Percentage difference in mean organ dose and standard deviation for a cardiac CT scan at 120 kV with tube current modulation of: C1–A cardiac CT using the cardiac voxel model compared to a cardiac CT using the same model but taking into account the entire organ volume, C2–A cardiac CT using the cardiac voxel model but taking into account the entire organ volume compared to a cardiac CT with overscan using the whole-body voxel model, C3–A cardiac CT with overscan compared to a cardiac CT without overscan using the whole-body voxel model, C4–A cardiac CT using the cardiac voxel model compared to a cardiac CT with overscan using the whole-body voxel model

	Organ dose difference (%)			
Organ	C1	C2	C3	C4
Heart	0.0 ± 0.0	−8.1 ± 2.5	2.1 ± 0.9	−8.1 ± 2.5
Lungs	48.7 ± 12.7	−25.3 ± 4.2	6.5 ± 1.5	10.9 ± 8.5
Oesophagus	122.0 ± 254.7	−31.3 ± 9.7	6.8 ± 2.5	31.3 ± 20.2
Breast	16.3 ± 20.8	−12.8 ± 9.7	5.1 ± 3.1	−0.1 ± 8.9
Thyroid	−	−100.0 ± 0.0	12.8 ± 2.3	−100.0 ± 0.0
Ribs	92.2 ± 15.2	−30.7 ± 5.0	7.3 ± 2.9	32.7 ± 7.6
Liver	168.7 ± 125.9	−45.6 ± 10.8	10.1 ± 8.7	35.5 ± 26.9
Spine	432.1 ± 83.4	−41.5 ± 4.7	8.1 ± 3.7	208.3 ± 33.1
Kidneys	11,975.6 ± 21,386.2	−89.9 ± 7.0	16.2 ± 14.7	237.5 ± 95.5

### Influence of overscan

In standard clinical practice, most CT scans are performed helically which introduces the concept of overscan or overranging. Table 4–C3 and Table 5–C3 present the percentage difference in mean organ doses for a chest and cardiac CT performed with and without overscan using the whole-body voxel models. As expected, overscan induces higher organ doses. For chest CT, dose increases within 6% are observed for all organs except the kidneys. For the kidneys, an increase in dose of 12% is seen. For cardiac CT, dose differences are within 16% for all organs.

### The use of references organ volumes

**Fig. 4:**
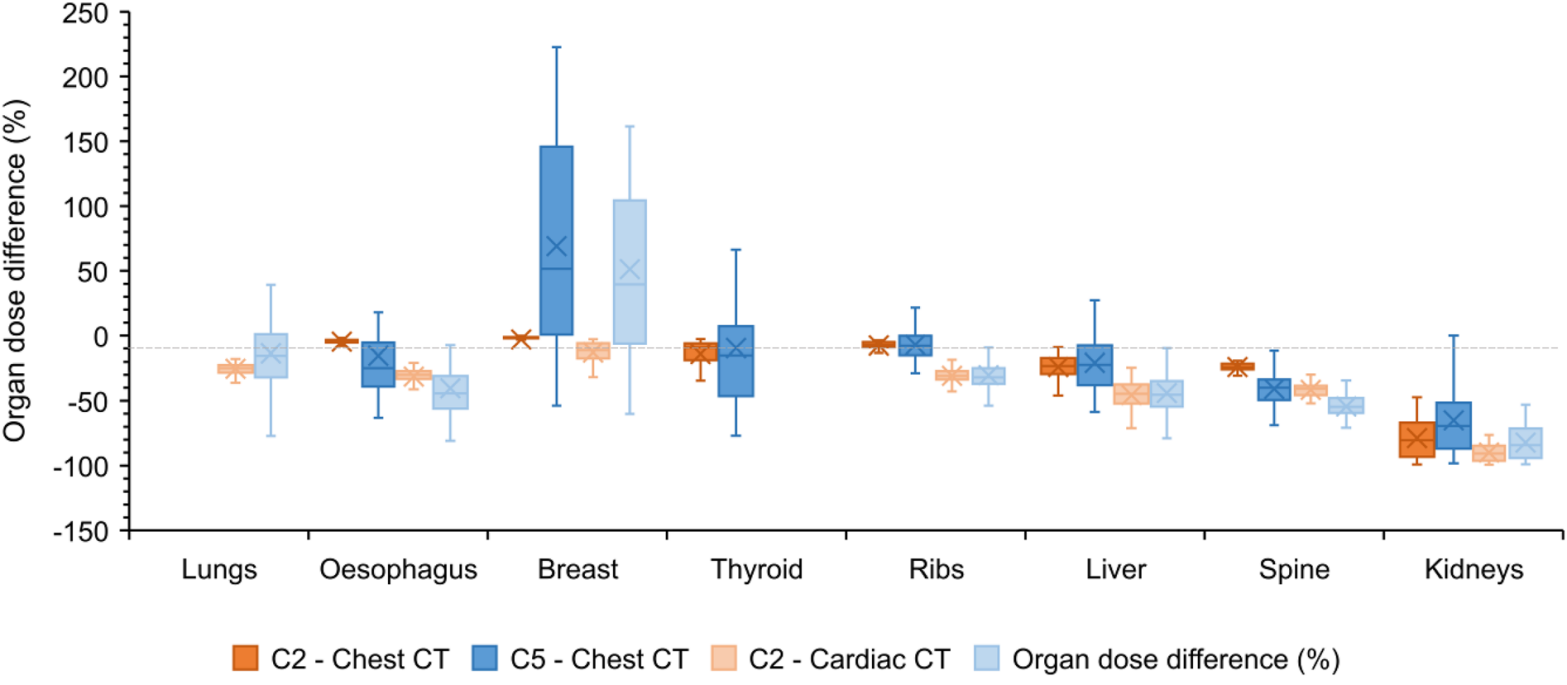
Distribution of the percentage difference in organ dose of organs partially in the field of view for a chest/cardiac CT scan performed with the thoracic/cardiac voxel model considering the real (C2) or ICRP reference (C5) organ volumes compared to a chest/cardiac CT with overscan using the whole-body voxel model

Figure 4 shows the distribution of the differences in organ doses between using the real or reference organ volumes in combination with the anatomy-specific voxel model, and the whole-body voxel model with incorporation of overscan for both chest and cardiac CT. A large spread in organ dose differences is seen when applying the reference volumes. For the thyroid, ribs and liver, good agreement in mean dose difference is found. The oesophagus and spine dose are more underestimated when the reference organ volumes are used while it is the opposite for the kidneys. In cardiac CT, the lung dose is less underestimated when applying the reference volume. For the breast, on the other hand, a considerable overestimation in CT dose is observed when applying the reference breast volume.

## Discussion

In daily routine, only CT images from the patient’s scanned body region are generated. Care must be taken when making conclusions about organ doses obtained through Monte Carlo simulations based on this limited patient data. Especially for organs lying only partially in the field of view special attention is needed. Although studies acknowledge the limitations of a voxel model limited to the clinical CT scan range, almost none of them studied the accuracy of organ dose estimations obtained with it. Franck et al. [8] solely looked at the influence of the applied paediatric voxel model on the accuracy of blood dose calculations while using the whole-body data to correlate organ doses with the size-specific dose estimate (SSDE). To our knowledge only Papadakis et al. [37] studied the difference in calculated organ doses originating from Monte Carlo simulations performed with a whole-body and corresponding anatomy-specific voxel model. Dose differences within 5% were found for all organs. However, these results are for a single patient, a 16-year-old boy, because no other paediatric whole-body CT images were available. Moreover, the MC simulations applied a fixed tube current.

In this study, the accuracy of patient-specific organ doses obtained through Monte Carlo simulations using anatomy-specific voxel models limited to the clinical CT scan range was estimated. Normalised organ doses were calculated for chest and cardiac CT scans of 50 adult patients simulated using both the thoracic or cardiac voxel model and the whole-body voxel model. The study population consisted of an equal number of male and female patients covering a wide BMI range. For all organs, little to no correlation (0 ≤ R^2^ < 0.3) was found between the contribution of scatter and patient characteristics, such as BMI and water equivalent diameter.

For organs covered entirely by the field of view, organ doses are only slightly underestimated when using the anatomy-specific voxel model. Because there is no information available outside the patient’s scan range, scatter contribution from the rest of the body as well as overscanning cannot accurately be taken into account. However, these underestimations are rather small. For chest CT dose underestimations within 7% were found for the breast, heart, lung, thyroid and oesophagus while for cardiac CT examinations an underestimation of around 8% was observed for the heart dose. Even though scatter radiation and overscan also contribute to the dose of organs lying more outside the field of view, an overestimation in organ dose is seen when the anatomy-specific voxel model is used. As observed, no dose differences were found for organs lying completely in the field of view when considering the entire organ volume in combination with the anatomy-specific voxel model while small to large dose differences were found for organs lying partially outside the field of view (Table 4–C1 and Table 5–C1). These differences become larger when in percentage terms more of the organ volume is situated outside the field of view. Although also the contribution of scatter radiation increases with a larger percentage of the organ volume located outside the field of view, it does not compensate for the dose decrease related to the knowledge of the entire organ volume. The observed overestimation in CT dose for organs lying partially outside the field of view is thus mainly due to the lack of information on the entire organ volume and increases with the percentage of the organ volume located outside the field of view (Table 4–C4 and Table 5–C4). This is found for the chest CT scans as well as for the simulated cardiac CT scans. The small dose increase observed for the lungs, which are completely covered by the CT scan range, relates to the amount of scatter radiation from the rest of the body incorporated in the last voxel model. The organ dose decrease observed for the liver and kidneys corresponds to the part of the organ lying outside the field of view. In contrast to the liver, which is half outside the field of view, the majority of the kidney volume was located outside the reconstructed scan range. This explains the larger dose decrease for the kidneys when using a whole-body voxel model. For organs located completely outside the field of view, such as the thyroid in cardiac CT, rather small radiation doses related to scatter are received by these organs. For example, for a cardiac CT performed at 112 mAs, scatter results into a thyroid dose of 1.0 ± 0.2 mGy.

Using voxel models generated from clinical CT image data has as limitation that they do not include the overscan in the z-direction. In this study, the thoracic and cardiac voxel models were created from whole-body PET/CT data. Therefore all necessary organ volumes were known and scatter from the rest of the body was incorporated in the Monte Carlo simulations as well. This allowed us to study the effect of overscan solely. As expected, overscan induces higher organ doses. The higher dose increase observed for the kidneys in chest CT, 12% compared to within 6% for all other organs, primarily originates from the fact that in percentage terms more of the organ is now irradiated. However, this increase of 12% is still relatively small. For a chest CT at 141 mAs this means an increase in dose from 3.7 ± 1.4 mGy towards 4.1 ± 1.7 mGy. Similar results were found for cardiac CT. In general, the amount of overscan is determined by the beam collimation, reconstruction slice thickness and pitch [38]. Tzedakis et al. [15] found that normalised effective dose values increased linearly with increasing z overscanning. The observed increase in organ doses could thus be larger when the overscan increases.

As can be seen in Tables 4 and 5, some standard deviations are larger than the observed mean percentage dose difference. For the breast, this is related to one specific patient. Small differences in organ dose together with the accuracy of the simulation software may explain the occasionally large standard deviations found between Monte Carlo simulations performed with and without inclusion of overscan using the whole-body voxel model (Table 4–C3). The larger standard deviations found for the kidneys and oesophagus, when comparing the doses obtained using the anatomy-specify voxel model with or without taking into account the entire organ volume, may be related to the percentage of the organ lying in the field of view. For the thyroid, it may be explained by its small size and superficial location.

When patient data is limited to the CT scan range, organ dose estimations are thus more accurate for organs located (almost) completely in the field of view. As shown in this study, the ICRP reference organ masses for the reference adult male and female may be a solution to compensate for the lack of information on the volume of organs lying partially outside the field of view [32–34]. Although for most organs little to no correlation (0 ≤ R^2^ < 0.3) was found between the real organ volume and patient characteristics, such as BMI, weight and water equivalent diameter, using the ICRP reference volumes results in a wider spread of estimated radiation doses for all organs. However, for organs like the thyroid, ribs and liver good agreement was found between the mean organ doses obtained using the real or reference organ volumes. The larger underestimation of the oesophagus dose may be related to differences in the length and diameter of the oesophagus between our study population and the reference adult male and female. According to ICRP Publication 89 the length varies generally in the range of 23–30 cm in adult males and 20–26 cm in adult females while the diameter has been estimated as 13–19 mm and 16–22 mm at the constrictions and dilated segments, respectively [32]. Differences in the length of the spine may also explain the observed larger underestimation of the organ dose. The observed overestimation of the breast dose when assuming the reference breast mass in the dose calculation must be taken with caution. Because each woman’s breast is different, using the reference breast volume may result in dose values with significant bias. This is reflected in the observed weak to moderate correlation (0.35 ≤ R^2^ < 0.55) between the real breast volume and patient characteristics like BMI, weight and water equivalent diameter.

Another approach may be the use of so-called hybrid computational phantoms to extend the patient’s anatomy. These third-generation hybrid phantoms provide the best features of stylised (first-generation) and voxel (second-generation) phantoms. Using non-uniform rational b-spline (NURBS) and polygon mesh (PM) surfaces to describe anatomical structures, they allow flexibility in fitting the phantom to patient anatomic data such as the body size and organ position [39, 40]. Some examples are the XCAT (extended Cardia-Torso) series [41], based on the Visible Male and Female anatomical datasets from the National Library of Medicine, and UF/NCI series [39, 42] developed at the University of Florida and National Cancer Institute. Beyond registering virtual phantoms to the actual scan range, other approaches exist ranging from simple ones like repeating the end slices towards complex methods such as AI synthesis.

To correct simulated organ doses for missing organ volume, scatter and overscan one approach could be the use of correction factors. Preferably, these are established based on a large population of patient data. As for the organ volume lying in the field of view, correction factors for scatter and overscan should be defined for each organ separately. However, we need to keep in mind that these correction factors are not patient-specific but relate to the mean of the population. For scatter and overscan, the established correction factors will probably be suitable for the majority of patients while this will not be the case for those defined to correct for organ volume outside the field of view. As was seen during this study, the organ volume located inside the field of view may vary strongly from patient to patient, especially for organs of which the majority of the volume is located outside the reconstructed scan range.

The development of automatic segmentation tools such as TotalSegmentator [43] and advances in deep learning create a lot of opportunities for future work. They make organ segmentation less time-consuming, which means that this study could be performed for more organs and a larger population of patients. Deep learning also opens the door towards a better correction for organ volume outside the field of view, scatter and overscan. Additionally, the estimated organ doses could be used to calculate the effective dose of a population. For partially irradiated organs it is beneficial to first correct for the missing organ volume. Because the radiation weighting factor for photons is equal to one, we then only have to take into account the different tissue weighting factors as defined in ICRP Publication 103 [44]. The effective dose is then defined as the weighted sum of the organ dose multiplied with tissue weighting factors.

## Conclusion

When the 3D patient model is limited to the anatomy-specific CT scan range, CT organ doses from Monte Carlo simulations are the most accurate for organs entirely in the field of view. For these organs only the radiation dose related to scatter from the rest of the body is not incorporated. For organs lying partially outside the field of view, organ doses are overestimated. This overestimation depends strongly on the amount of the organ volume located outside the field of view. Except for the breast, using the ICRP reference organ masses may result in more accurate dose estimations for these organs. As expected, overscan induces slightly higher organ doses.

## Data Availability

The authors declare that all data supporting the findings of this study are available within the article.
